# Ulcerative colitis progression: a retrospective analysis of disease burden using electronic medical records

**DOI:** 10.48101/ujms.v127.8833

**Published:** 2022-10-18

**Authors:** David Dahlgren, Lars Agréus, Jan Stålhammar, Per M. Hellström

**Affiliations:** a Department of Pharmaceutical Biosciences, Translational Drug Discovery and Development, Uppsala University, Uppsala, Sweden; b Department of Neurobiology, Care Sciences and Society, Karolinska Institutet, Stockholm, Sweden; c Department of Public Health and Caring Sciences, Family Medicine and Preventive Medicine, Uppsala University, Uppsala, Sweden; d Department of Medical Sciences, Uppsala University, Uppsala, Sweden

**Keywords:** Ulcerative colitis, health economics, inflammation, outcomes research, inflammatory bowel disease

## Abstract

**Background:**

Ulcerative colitis (UC) is a debilitating inflammatory bowel disease. Present knowledge regarding UC disease progression over time is limited.

**Objective:**

To assess UC progression to severe disease along with disease burden and associated factors.

**Methods:**

Electronic medical records linked with Swedish national health registries (2005–2015) were used to identify disease progression of UC. Odds of all-cause and disease-related hospitalization within 1 year were compared between patients with disease progression and those without. Annual indirect costs were calculated based on sick leave, and factors related to UC progression were examined.

**Results:**

Of the 1,361 patients with moderate UC, 24% progressed to severe disease during a median of 5.2 years. Severe UC had significantly higher odds for all-cause (OR [odds ratio] 1.47, 95% CI [confidence interval]: 1.12–1.94, *P* < 0.01) and UC-related hospitalization (OR 2.47, 95% CI: 1.76–3.47, *P* < 0.0001) compared to moderate disease. Average sick leave was higher in patients who progressed compared to those who did not (64.4 vs 38.6 days, *P* < 0.001), with higher indirect costs of 151,800 SEK (16,415 €) compared with 92,839 SEK (10,039 €) (*P* < 0.001), respectively. UC progression was related to young age (OR 1.62, 95% CI: 1.17–2.25, *P* < 0.01), long disease duration (OR 1.09, 95% CI: 1.03–1.15, *P* < 0.001), and use of corticosteroids (OR 2.49, 95% CI: 1.67–3.72, *P* < 0.001).

**Conclusion:**

Disease progression from moderate to severe UC is associated with more frequent and longer hospitalizations and sick leave. Patients at young age with long disease duration and more frequent glucocorticosteroid medication are associated with progression to severe UC.

## Introduction

Ulcerative colitis (UC) is a chronic inflammatory bowel disease (IBD) with a relapsing-remitting course of disease activity of the colon (1). The incidence of UC in the Uppsala Region, Sweden, is reported to 20 cases per 100,000 inhabitants (2). UC is a debilitating disease with unpredictable course, which is severely impacting on patients’ health-related quality of life (3–6).

UC is limited to the colon. The treatment is based on which part of the colon that is affected as well as the severity of the disease activity (1, 7). The pharmacological treatment options are based on 5-aminosalicylates, glucocorticosteroids, various immunosuppressants, and targeted immunomodulators (8), whereas surgical colectomy today is seen as a rescue in medically treatment resistant cases. The primary goal of therapy is to attain clinical remission, defined as symptomatic remission with no remaining glucocorticosteroid therapy. Despite a plethora of treatment options, there are unmet medical needs in UC, particularly regarding the impact of UC and its treatment in activities of daily living (4, 9). UC has an onset often in adolescence or early adulthood resulting in sizeable long-term medical healthcare, reduced quality of life, and ability to work (5, 10–12). Now, confirmative data delineating the progressive nature of UC are limited as well as outcomes associated with UC disease progression.

The primary objective of this study was to assess progression rate from moderate to severe UC, and the secondary objective was to investigate the medical healthcare resource burden associated with disease progression and to identify factors connected to disease progression in a cohort of Swedish UC patients.

## Materials and methods

### Study design

This study data were retrieved from electronic medical records (EMRs) in Uppsala County Council, Sweden. EMRs were cross analyzed with data from the Swedish National Patient Registry, Cause of Death Registry, Prescribed Drug Registry, and National Socioeconomic Registry. Data were pseudonymized as regards age, sex, prescriptions, diagnoses, laboratory test results, referrals, and clinical variables as retrieved from EMRs in primary care and secondary care settings. This study complies with applicable laws, regulations, and guidance regarding patient protection, including privacy. This study was approved by the Uppsala Ethics Review Board and filed under PYG-998351, November 28, 2014.

### Data collection

The primary data source was EMRs from primary and secondary care centers/hospitals in Uppsala County Council. Data from approximately 30 primary care centers in the Uppsala County Council including two hospitals (Enköping, Uppsala) were collected. In a few cases, the same variables were collected from EMR and one of the health registries. If data then deviated, the EMR database and Prescription Registry were considered gold standards for outcomes and prescribed medications, respectively. The primary source EMR data were linked to the different health registries through the individual Swedish personal identity number (PIN), which is unique for each Swedish citizen. Extracted EMR data were stored in a key code file retaining the possibility for data verification, and an analysis file for statistical operations. Thus, personal data in the analysis file were pseudonymized by replacing the PIN with a unique internal patient identification number. For posterity, the key code file is safely preserved, accessible only for the principal investigator.

Patients with a diagnosis of UC (K51) were identified from the EMR using the Pygargus Customized eXtraction Program (CXP, version 4.0) data extraction tool. The CXP has been used since 2005 in Swedish and Norwegian healthcare systems and in several previously published research studies with high accuracy (13–15). The integrated EMR data of Uppsala County Council allow for retrospective data capture over at least 7–8 years from primary care and secondary hospital care.

Patient data from the Swedish National Patient Registry were collected from inpatient and outpatient specialist medical care across all of Sweden. Key variables collected include diagnosis, surgery, sex, age, region, hospital visits, gastroenterology surgery specialist visits, as well as hospital admissions and discharges. Detailed information is available on all medical procedures and surgeries performed in both inpatient and outpatient settings.

The Cause of Death Registry contains information on the death cause of all Swedish residents and non-Swedish citizens living in Sweden, as well as Swedish citizen whose deaths did not occur in Sweden. The main variables in the registry include personal identification numbers; home districts; sex; date of death; underlying cause of death; nature of injury; multiple causes of death; and if the death was alcohol-, narcotic-, or diabetes-related. In the present study, the Cause of Death Registry was used to censor patients who died during follow-up. The censoring date was set to January 1, 2015.

Data on prescribed drugs were obtained from EMR supplemented by linking individual patient identifiers to the National Prescribed Drug Registry and were used to identify the total IBD population. The National Prescribed Drug Registry contains data on all prescription drugs dispensed at Swedish pharmacies (16). The registry covers prescriptions from both primary and specialist care level and includes data on prescription date, dosage, pack size, healthcare drug prescriber, and costs associated with the drug prescription. In our study, prescription data were retrieved from EMR as well as the National Prescribed Drug Registry. The difference between the two sources is that prescription data from EMR include prescribed medications, whereas the National Prescribed Drug Registry records dispensed medications. Drugs administered during hospitalization are recorded in EMR.

The National Socioeconomic Registry includes information on all individuals over 16 years of age registered in Sweden. This longitudinal database integrates information on educational level, marital status and family situation, occupational status, retirement, economic compensation, and social benefits. Our study obtained information from this registry on unemployment, sick leave, early retirement, and disability.

### Study population and follow-up

Patients of 18 years or older with a UC diagnosis from July 2005 until January 2015 were identified using *International Statistical Classification of Diseases and Related Health Problems, 10th Revision* codes (K51 UC including all subdiagnoses: K51.0 ulcerative [chronic] pancolitis, K51.2 ulcerative proctitis, K51.3 ulcerative proctosigmoiditis, K51.4 pseudopolyps of colon, K51.5 left-sided colitis, K51.8 other specified UC, K51.9 UC, unspecified). Moderate disease activity was defined as having a partial Mayo score of 2–4 being treated with prednisolone <40 mg/day or its equivalent. Severe disease was defined as having a partial Mayo score of 5–9 and being treated with prednisone ≥40 mg/day or its equivalent. Patients were followed from the date of eligibility with moderate UC until the date of progression to severe UC.

### Outcomes

The rate of disease progression from moderate to severe UC was determined over a 5.2-year median follow-up period. Factors associated with disease progression were measured 1 year prior to index date, including age, sex, disease duration, medication use (e.g., corticosteroid and 5-aminosalicylic acid), and serum C-reactive protein (S-CRP) levels. Index date for patients was defined as the date of progression from partial Mayo score 2–4 to score 5–9. For patients who did not progress, the index date was the earliest date qualifying as moderate UC.

Over the median 5.2-year follow-up, the burden of healthcare resource utilization was assessed by determining the presence of all-cause hospitalization, UC-related hospitalization, and UC-related surgery within 1 year before and after the index date among patients who progressed to severe UC and those who did not progress. The average annual number of sick leave days and the annual indirect costs associated with sick leaves were calculated for both cohorts in patients aged 18–65 years.

### Statistical data analyses

Statistical Analysis Software, version 9.3 (SAS Institute, Cary, NC, USA), was used for data management and statistical analyses. For the primary endpoint, Kaplan–Meier analysis was used to depict the progression rate from moderate to severe UC. The secondary endpoints including demographic and clinical factors associated with disease progression were analyzed with a logistic regression model. Frequency and proportion of elevated serum CRP (>30 mg/L) and fecal calprotectin (>50 mg/g) were compared between the two groups. For patients whose disease progressed versus those patients who did not progress, risks of all-cause hospitalization, UC-related hospitalization, and UC-related surgery were assessed as dependent variables using multivariate logistic regression models controlling for age, gender, and duration of disease; baseline corticosteroid and 5-aminosalicylic acid use; and baseline all-cause hospitalization. Odds ratios (OR) with 95% confidence intervals (CI) were estimated from logistic regression models.

Indirect costs were calculated within 2 years after patients entered a moderate disease condition based on annual number of sick leave days. Indirect costs were calculated by multiplying the average annual number of sick leave days × average annual Swedish salary, adjusting for gender (assuming 250 work days per year). Annual sick leave days were obtained from the National Socioeconomic Registry. The average annual salary in 2014 was 350,400 SEK for women and 403,200 SEK for men (excluding social fees 47%) (17). Indirect costs were converted to 2020 value of SEK and reported in Euro (€) for comparison. Chi-square or Kruskal–Wallis tests were used for comparisons between patients with or without progressing disease.

## Results

### Demographics and clinical characteristics

In total, 2,450 patients diagnosed with UC (K51) were retrieved from the EMR over the period 2005–2015. The vast majority of the diagnoses were made at gastroenterology, internal medicine, and surgery secondary care units and only few at other care units. Largely, 60% of patients were classified as unspecified UC according to the ICD-10, the majority of which with moderate disease activity. Of all UC patients registered in the EMR, 9% were not retrievable through extraction with the CXP 4.0 (Table 1). There were also numerous comorbidities with arthritis, asthma bronchiale, and cancer being the most prevalent; however, none of which exceeding 8% in the study cohort (Table 2).

**Table 1:** Demographics, diagnosis setting, and clinical characteristics of study cohort with UC extracted from electronic medical records by the customized extraction program, version 4.0.

Variable	All UC patients, *N* = 2,450 (%)
Gender	
Female	1,185 (48.4)
Male	1,265 (51.6)
Diagnosed with UC, *N* (%)	
Missing	231
Gastroenterology care unit	633 (28.5)
Internal medicine care unit	415 (18.7)
Other specialist unit	105 (4.7)
Pediatric care unit	140 (6.3)
Surgery care unit	926 (41.7)
Disease extension	
Ulcerative proctitis	354 (14.6)
Left-sided colitis	205 (8.5)
Extensive colitis	400 (16.5)
Unspecified UC	1,491 (60.2)
Disease severity	
Mild	668 (27.2)
Moderate	1,361 (55.6)
Severe	421 (17.2)
Medical treatment	
Mesalazine	2,352 (96.0)
Glucocorticosteroids	1,788 (72.9)
Immunomodulators	613 (25.0)
Antitumor necrosis factor-α	318 (12.9)

UC, ulcerative colitis.

**Table 2:** Number of comorbidities to UC in the study cohort.

Comorbidities	Number (% of cohort)
Anxiety	140 (6.0)
Arthritis	175 (7.7)
Asthma bronchiale	181 (7.7)
Bronchitis	6 (0.3)
Cancer	139 (6.2)
Chronic obstructive pulmonary disorder	38 (1.6)
Depression	41 (1.7)
Diabetes mellitus	133 (5.6)
Hyperthyroidism	22 (0.9)
Hypothyroidism	53 (2.2)
Malnutrition	1 (0.04)
Renal insufficiency	4 (0.16)
Multiple sclerosis	10 (0.4)
Myasthenia gravis	1 (0.04)
Osteoporosis	25 (1)
Primary sclerosing cholangitis	73 (3.1)
Psoriasis	61 (2.5)
Pyoderma gangrenosum	7 (0.3)
Rheumatoid arthritis	15 (0.6)
Thromboembolism	69 (2.9)
Thyroiditis	7 (0.3)
Uveitis	49 (2)

### Rate of disease progression and associated factors

**Figure 1:**
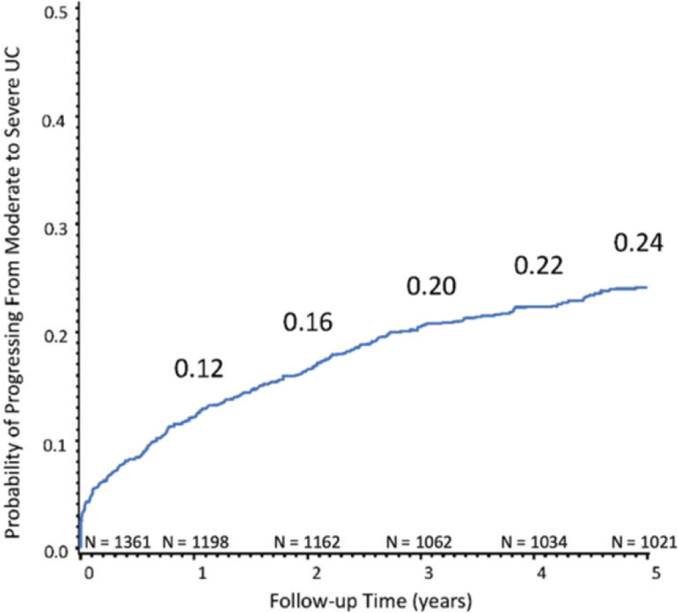
Probability of progressing from moderate to severe ulcerative colitis over the median 5.2-year follow-up. The numbers at risk are given for each time point of interest. UC, ulcerative colitis.

Out of 1,361 patients identified with moderate UC in the EMR, 321 (24%) developed severe UC over a median follow-up period of 5.2 years (interquartile range: 4.8 years) (Figure 1). During the follow-up, 121 of the 1,361 (9%) patients used biologic therapies, more specifically 48 out of the 321 (15%) patients in the progression group and 73 of 1,040 (7%) among those in the non-progression group.

The odds for progression from moderate to severe UC were 1.62 (95% CI: 1.17 to 2.25, *P* < 0.01) times greater in patients below 30 years of age and 1.38 (95% CI: 1.01 to 1.90, *P* = 0.04) times greater in patients between 30 and 50 years of age compared with those who were aged over 50 years (Table 3). In addition, glucocorticosteroid use (OR 2.49, 95% CI: 1.67 to 3.72, *P* < 0.0001), elevated S-CRP (OR 1.59, 95% CI: 1.21 to 2.08, *P* < 0.01), and long disease duration (OR 1.09, 95% CI: 1.03 to 1.15, *P* < 0.01) were associated with significantly greater odds for progression from moderate to severe disease.

**Table 3:** Demographic and baseline clinical factors associated with progression from moderate to severe UC over a median 5.2-year follow-up.

Variable	No progression (*n* = 1,040)	Progression (*n* = 321)	Odds ratio (95% CI)	*P* value
Aged <30 years, *n* (%)	276 (26.5)	104 (32.4)	1.62 (1.17–2.25)	<0.01
Aged 30–50 years, *n* (%)	355 (34.1)	115 (35.8)	1.38 (1.01–1.90)	0.04
Aged >50 years, *n* (%)	409 (39.3)	102 (31.8)	1.00 (reference)	–
Female, *n* (%)	488 (46.9)	155 (48.3)	1.16 (0.90–1.51)	0.26
Male, *n* (%)	552 (53.1)	166 (51.7)	1.00 (reference)	–
UC disease duration, y (mean ± SD)	4.9 ± 2.7	5.8 ± 2.6	1.09 (1.03–1.15)	<0.01
Glucocorticosteroids, *n* (%)	867 (83.4)	316 (98.4)	2.49 (1.67–3.72)	<0.0001
5-ASA, *n* (%)	489 (47.0)	155 (48.3)	1.25 (0.95–1.65)	0.11
CRP: elevated^a^, *n* (%)	362 (34.8)	161 (50.2)	1.59 (1.21–2.08)	<0.01
CRP: not elevated, *n* (%)	492 (47.3)	141 (43.9)	1.00 (reference)	–

5-ASA, 5-aminosalicylic acid; CI, confidence interval; CRP, C-reactive protein; SD, standard deviation; UC, ulcerative colitis; y, years.

a Elevated CRP was defined as >30 mg/L.

### Healthcare resource utilization and work productivity associated with disease progression

**Figure 2:**
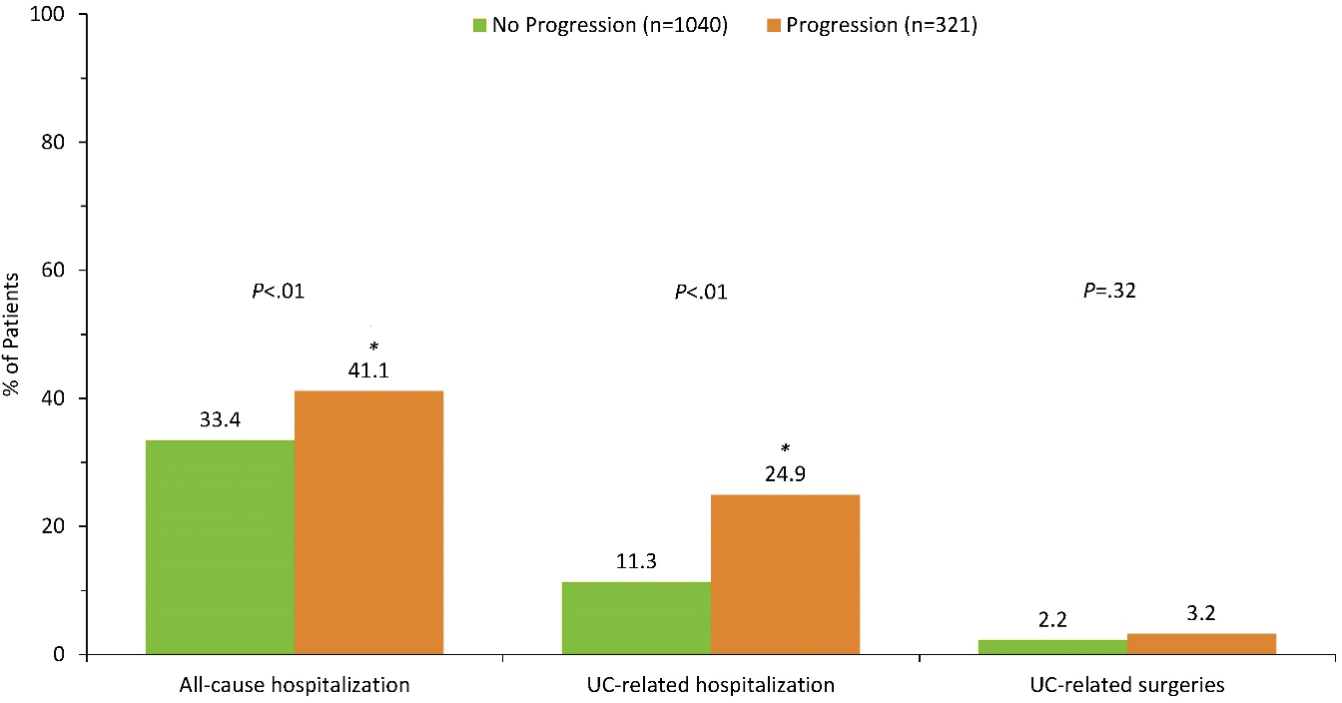
Disease burden in patients progressing from moderate to severe ulcerative colitis. UC, ulcerative colitis.

Over the 5 years of follow-up, higher rate of all-cause hospitalization (41% vs 33%, *P* < 0.01) and UC-related hospitalization (25% vs 11%, *P* < 0.01) were found in the patient group who progressed to severe UC as compared to those who did not progress (Figure 2). Among patients with disease progression to severe UC, the odds were 1.47 times greater for all-cause hospitalization (95% CI: 1.12 to 1.94, *P* < 0.01) and 2.47 times greater for UC-related hospitalization (95% CI: 1.76 to 3.47, *P* < 0.001). There was no difference in UC-related surgery between the two groups.

**Figure 3:**
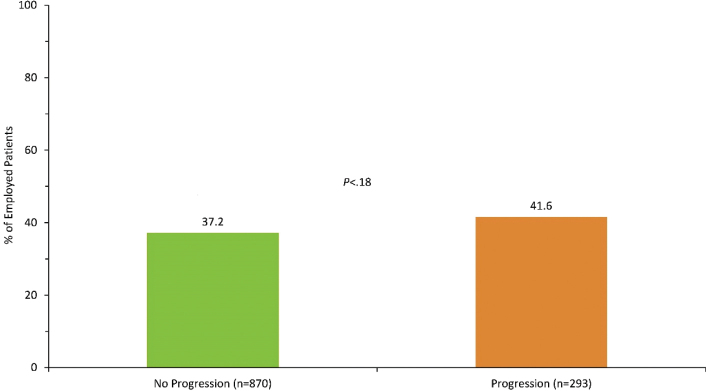
Proportion of unemployed patients.

**Figure 4:**
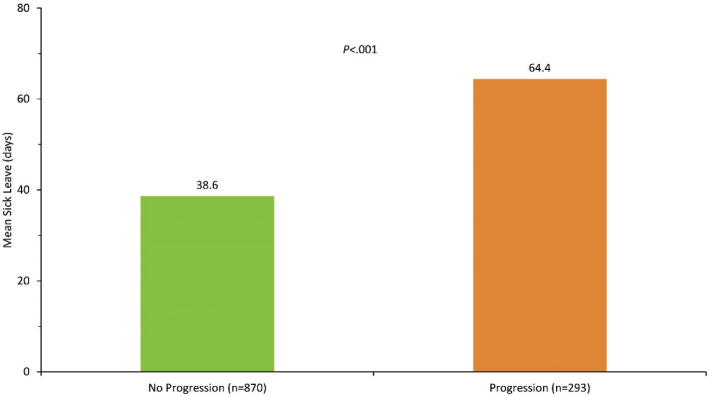
Sick leave within 1 year.

**Figure 5:**
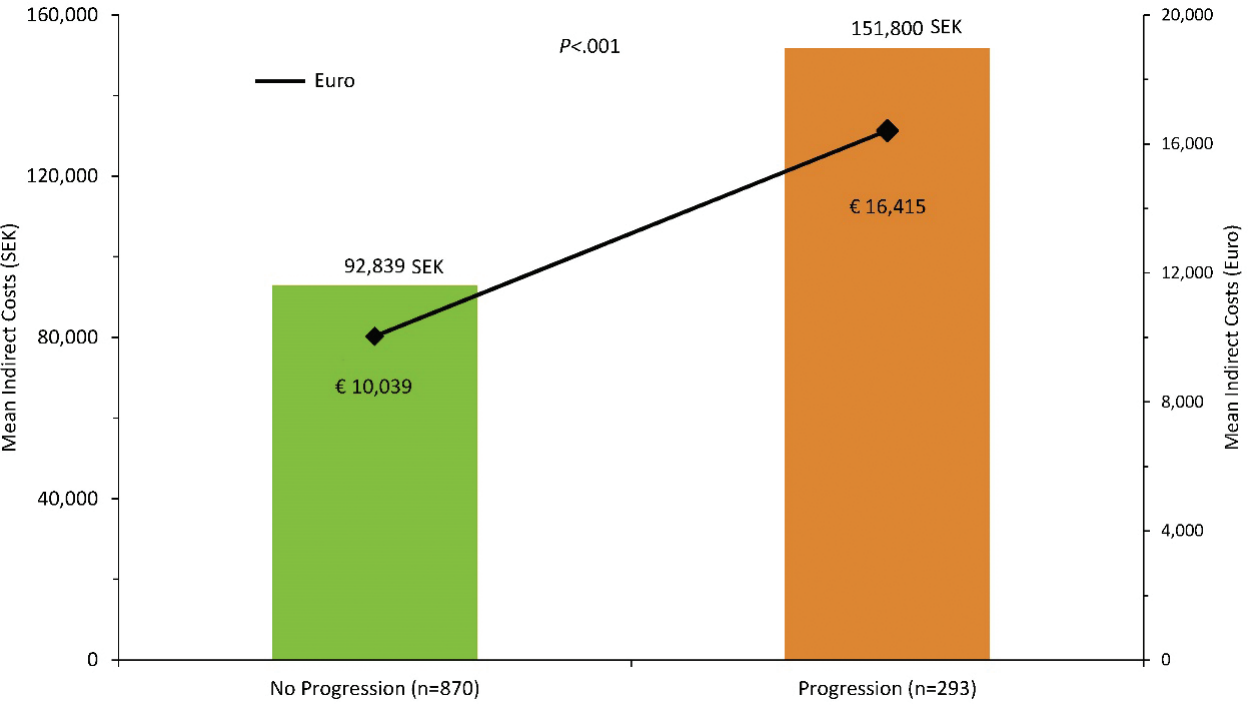
Costs associated with sick leave. SEK, Swedish krona; €, Euro.

Unemployment among those progressing to severe UC was only numerically greater than for those who did not (42% vs 37%, *P* = 0.18; Figure 3), while the average sick leave days were significantly higher among patients with progressive disease (64% vs 39%, *P* < 0.001; Figure 4). The higher frequency of sick leave days in the progression group translated to higher indirect costs with 151,800 SEK (16,415 €) vs 92,839 SEK (10,039 €) compared with the non-progression group (*P* < 0.001; Figure 5).

## Discussion

Our present study investigated the rate of progression from moderate to severe UC in a cohort of Swedish patients 2005–2015. EMR data linked to multiple national registries were analyzed in order to reflect real-world practice in patients with UC. Using the ICD classification of diagnoses, our study shows that approximately one-fourth of patients with moderate UC progress to severe UC over a period of 5 years. The odds of a patient experiencing a UC-related hospitalization were two-fold greater in those with progressive disease resulting in a sizeable medical and societal burden; specifically, the loss of work productivity results from absenteeism. Logistic regression analysis of factors associated with the disease indicates that younger age, long disease duration, and glucocorticosteroid use were associated with progression to severe UC.

The disease progression rate in our present study is in agreement with the disease extension rate reported in a recent meta-analysis (18). Results of the meta-analysis showed that the rate of disease extension from proctitis or left-sided colitis to extensive UC was 18% at 5 years and 31% at 10 years (18). Furthermore, in three different population-based cohort studies, 21–24% of UC patients showed progressive disease from limited to extensive colitis, and 5–10% underwent a colectomy during a forthcoming 5- to 10-year period (19–21). Several meta-analyses (22–25) report an increased risk of colitis-associated colorectal cancer in patients with UC compared to the general population. This increased risk has been found to be related to young age at disease onset, prolonged disease duration, extent of disease, and male sex. In our current study, young age, prolonged disease duration, and extended corticosteroid use were all found to be significant factors associated with disease progression. These results are in line with those reported for patients with chronic disease activity, frequent relapses, need for systemic glucocorticosteroid use, and young age at diagnosis (26–28). As shown in our study, these same factors are associated with considerable work disability (29).

UC is often diagnosed in adolescence or early adulthood in the working-age population, and substantial work productivity losses among patients with moderate to severe disease have been reported (5, 11, 29). Our study confirms that a greater proportion of patients with progressive disease were more frequently unemployed compared with those who had a non-progressive disease. Hence, the average annual number of sick leave days was significantly greater in those with progressive disease resulting in 1.6 times higher indirect costs in patients with progressive disease as compared to those maintaining moderate disease activity. To this end, the use of medical healthcare resources was found to be higher in patients with progressive UC as the prevalence of all-cause and UC-related hospitalization was significantly greater in this patient group. Though the overall disease progression rate may not be deemed to be aggressive, the cost of disease progression in UC is significant in terms of medical costs as well as societal burden.

Despite the availability of antibody-based biologics to treat UC during the study period, biologic use was only 9% overall in this UC population during a median of 5-year follow-up. Even among patients with aggressive disease activity progressing to severe UC, the use of biologics was 15%. The use of biologics reported in other population-based cohort studies with assessment of disease extension has also been relatively low ranging from 5 to 11% (20, 21, 30), consistent with the overall use of biologics. In agreement with this, the use of biologics, in general, was reported to be 7.4% in a recent study, estimating the annual societal cost of UC in Sweden (31). These findings suggest that patients with UC are being managed predominantly with 5-aminosalicylates and corticosteroids to induce remission and control symptoms against a suggested background treatment with azathioprine treatment in 23% of this disease population (20).

Guidelines for UC treatment support a treat-to-target approach, in which the therapeutic goal includes not only control of symptoms and improved quality of life but also the prevention of disease progression, bowel damage, surgery, and disability (32–36). The ultimate targets of treatment in UC remain to be defined but may include clinical and patient-related outcomes (PROs) such as resolution of rectal bleeding and normalization of bowel habits; endoscopic and histological mucosal healing; and normalization of biomarker targets such as serum CRP and fecal calprotectin levels. To accomplish treat-to-target goals, therapy needs to begin early, be aggressive and optimized, and then patients need to be monitored regularly until predefined response goals are achieved. Given the burden associated with UC progression, broader use of biologics earlier in the disease course is demanded to accomplish these treat-to-target goals.

One of the strengths of the current study is the linkage of several databases including the most proximal EMR to provide a comprehensive and robust evaluation of data in a large cohort of patients with moderate to severe UC (37). However, there are some limitations that need to be considered when interpreting the results of the present study. Although clinical outcomes reported as partial Mayo scores and the ICD classification are available, some clinical factors are not fully captured, such as endoscopic findings with disease location. Also, since the proportion of patients on biologics treatment was low and the proportion of patients with fecal calprotectin analysis was low (38% in progression group and 22% in non-progression group), these two factors were not included in the regression model. However, despite the low frequency of reported fecal calprotectin values, numerically more patients with UC progression compared with no progression had elevated fecal calprotectin, being 87% versus 79%.

In this Swedish UC population, progression of moderate to severe UC occurred in approximately one-fourth of the patients with moderate disease. The disease burden associated with hospitalizations and sick leave and their associated costs were significantly higher in patients who progressed to severe UC compared with those who did not. Longer disease duration, young age, glucocorticosteroid use, and elevated serum CRP levels were markedly associated with disease progression. Early identification of disease progression and intervention with appropriate effective treatment should be implemented early to prevent the considerable disease burden associated with UC progression.
